# Another Limping Child: An Interesting Diagnosis Journey

**Published:** 2017

**Authors:** Mehrnoush HASSAS YEGANEH, Khosro RAHMANI, Shokuh HASHEMI, Seyed Hassan TONEKABONI, Reza SINAE, Mohammad reza FATHI, Reza SHIARI

**Affiliations:** 1 Department of Pediatric Rheumatology, ShahidBeheshti University of Medical Sciences, Mofid Children’s Hospital, Tehran, Iran.; 2 ShahidBeheshti University of Medical Science, Tehran, Iran.; 3 Pediatric Neurology Research Center, Research Institute for Children Health, ShahidBeheshti University of Medical Sciences, Tehran, Iran.; 4 Pediatric Neurology Department, Mofid Children’s Hospital, Faculty of Medicine, ShahidBeheshti University of Medical Sciences, Tehran, Iran.

**Keywords:** limping child, Weakness, Hypercalciuria

## Abstract

Limp is described as any deviation from a normal gait pattern for the child’s age. Limping takes many forms and is one of the most enigmatic complaints in pediatric medicine. It is never normal, and both benign and life-threatening illnesses can present with limp. The provisional diagnosis can be a challenge to establish even after history, physical, and laboratory examinations.

## Introduction

Limping is a symptom of different diagnoses in children. In order to diagnose, a careful history and physical examination besides laboratory tests and imaging are necessary.

Idiopathic Hypercalciuria (IH) is a metabolic disorder which influences a variety of groups without considering the role of sex or race.

Hypercalciuria can be a risk factor for limping in childhood.

## Case Report

A 9 year - old professional athlete girl was brought to Mofid Children Hospital, Tehran, Iran in 2013 with limping. She complained of lower limb weakness, muscular spasmand pain which she could not localize and had lasted for about 5 months. She claimed that her pain was aggravated in mornings and with activity and did not ease with resting. She also reported recurrent vertebral, abdominal and right flank pain with transient dysuria. She had no history of dark or discolored urine or nephrolithiasis and had difficulty rising, standing and climbing stairs. According to her parents, there was no history of fever, trauma, drug exposure, animal bite, or recent Immunizations. She had no rashes and breathing or swallowing difficulty. There was no history of seizures or of eating canned food. Her parents reported a one-month long loss of appetite and a weight loss of about 9 k gain the last 4 months. Private interview with the patient revealed no abuse. Her family history consisted of frequent nephrolithiasis of his father.

She was alert, lying on her side and unwilling to walk. Her pulse rate was 120 beats per min; respiratory rate, 17breaths per min; and body temperature, 36.5 Celsius centigrade. No lymphadenopathy was found.

The neck was supple. The lungs and heart were normal. Her abdomen was soft and non-tender. There was no swelling or wasting of any muscle. Muscle force appeared normal except for the right lower limb which was diminished to 4/5^th^. Fasciculation, myoclonus, and muscular atrophy were not observed. The patient had no skin lesions, bruises or sacral dimple. Neurological examination was normal. Orthopedical examinations revealed an antalgic gait with a positive FABER test in right hip. Joint examinations showed no swelling, tenderness, warmth and restricted range of motion except for the right hip joint in which showed a restriction in flexion and lateral rotation.

Due to her high energy sport activities and a possibility of cervical spinal trauma, a cervical MRI and a pelvic plain radiograph was ordered. Cervical MRI showed Loss of normal cervical lordosis along with mild scoliosis which was convex toward the right side and minimal disc dehydration in some levels. Pelvic radiograph revealed no fracture or asymmetry.

Complete blood count showed white blood cells 7700/mm3 with normal differential count and hemoglobin of 11 g/dl with a MCV of 79, platelets 572000/mm3. Serum biochemistry tests revealed sodium, 138 mmol/L; potassium, 4.6 mmol/L; urea, 11mg/dL; creatinine, 0.7 mg/dL; glucose, 98 mg/dL; and calcium level, 8.5 mg/dL. Liver function profiles were normal. Her lactate dehydrogenase level was 294 U/L (105–215). Serum creatininekinase and aldolase levels were 58 U/L (40–210) and 26.4 U/L(1.2–8.8), respectively. Her urine analysis showed calcium oxalate crystals and calcium excretion in relation to creatinine in fasting urine was 0.5 which indicates hypercalciuria. Parathyroid hormone level was 7pg/ml Abdominal and pelvic ultrasonography was normal. Right hip joint ultrasonography showed no swelling or dislocation. To rule out myopathies, peripheral neuropathies and myositis, electromyography (EMG) and motor nerve conduction velocity (NCV) was ordered which were normal.

With the initial diagnosis of malignancy in mind, the patient underwent a bone survey in which lucent band in left distal femur and patchy sclerosis in distal femur and proximal tibia was noted. A decrease in height of anterior body of T12 vertebra and T11 collapse was significant. Diffuse osteopenia was noticed. Bone densitometry reported a T-Score of -2.31 in femoral neck which is compatible with severe Osteopenia. The BMD values in lumbar spine were also compatible with moderate osteopenia.

Further workup for metastatic bone involvement was done by a whole body bone scan which showed increased uptake in greater trochanter of right femur, right ischium, right S1 joint and T4 and T12 vertebras, left clavicle and sternum. At this point differential diagnosis included metastatic involvement, metabolic bone disease and multiple traumatic lesions. Abdominal and pelvic Computed tomography scan and chest x-ray was normal. Bone marrow aspiration and its immune phenotyping were normal. Vertebral needle biopsy reported no exudative inflammation, necrotizing granulomas or neoplasms.

**Figure 1:**
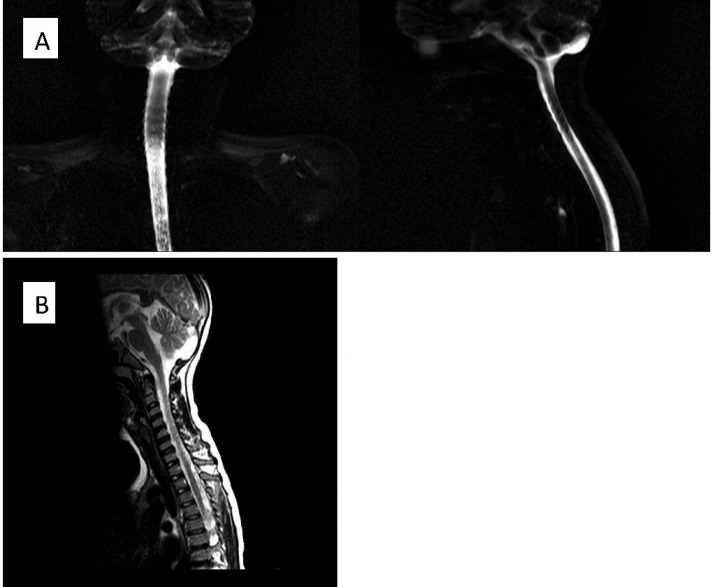
Cervical magnetic resonance imaging shows mild scoliosis and loss of cervical lordosis (A), No discopathy is notable (B).

**Figure 2:**
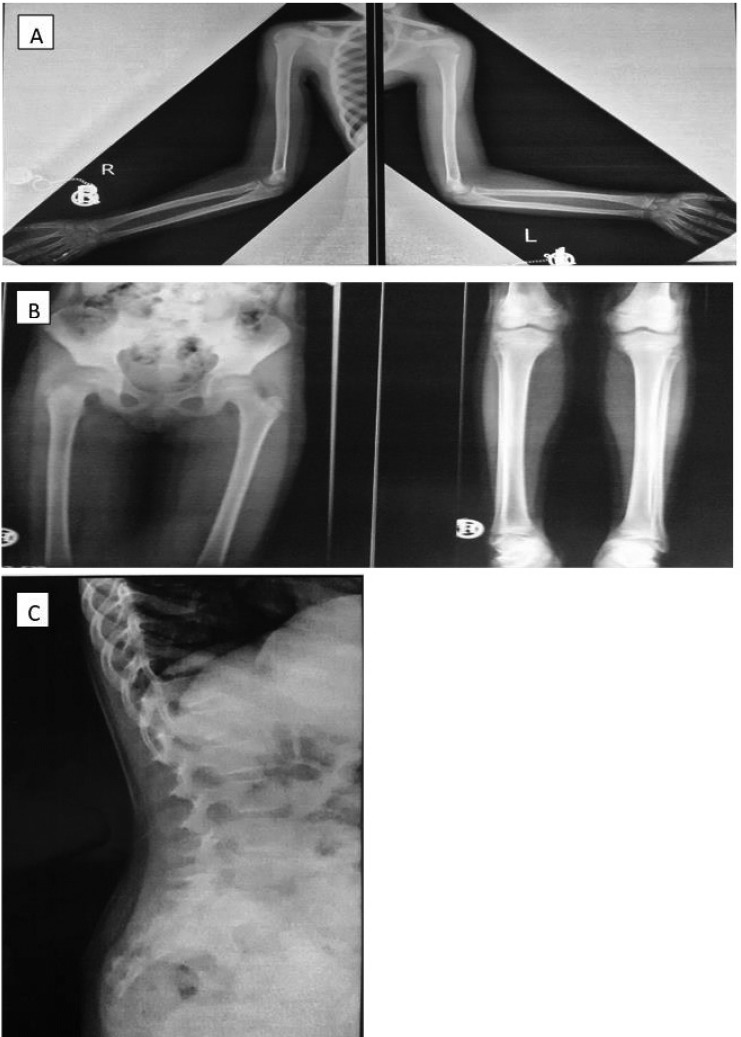
Bone survey shows diffuse osteopenia.lucent band in left distalfemur and patchy sclerosis in distal femur and proximal tibia are noted (B). Vertebralcollapse is present at Tll- T12 levels (C

**Figure 3:**
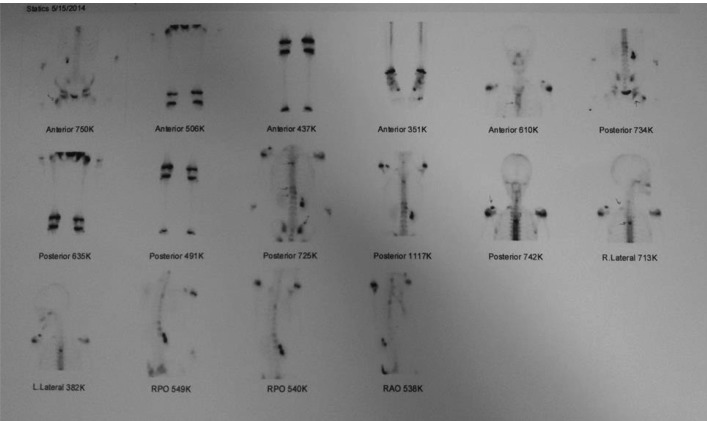
Whole body bone scan shows increased uptake in greater trochanter of right femur, right ischium, right S1 joint and T4 and T12 vertebras, left clavicle and sternum

According to the family history and her recurrent abdominal and flank pains and generalized weakness with frequent dysuria, steopenia and a normal parathyroid hormone level, the diagnosis of idiopathic hypercalciuria was made. All the patient’s sign and symptoms including generalized weakness and muscular spasms were compatible with the diagnosis.

She was subsequently prescribed thiazide diuretic and her symptoms improved gradually. She was discharged with an improved gait 3 days after starting thiazide and her BMD values were improved after 5 months.

## Discussion

Children may limp for many reasons. In order to diagnose properly, a thorough history and physical examination besides appropriate laboratory tests and imaging are necessary. 

We know that idiopathic hypercalciuria (IH) is a metabolic reformation which influences a variety of groups without considering the role of sex or race(1,2) and is a leading metabolic risk factor for urolithiasis and is the major metabolic disorder found in children(1, 3, 4).

The clinical presentation of IH includes gross or microscopic hematuria, voiding symptoms (urinary urgency, dysuria, incontinence, enuresis and suprapubic pain), urinary tract infection, as well as back, flank, or abdominal pain in the absence of urolithiasis and, less frequently, acute renal colic (3, 5, 6).

The ureter lies just medial to the psoas muscle. Thus, kidney stones passing ureter or in our case hypercalciuria causing recurrent flank colic pain irritates the psoas muscle, leading to psoas muscle syndrome. Just as in our case, it is postulated that psoas syndrome may frequently manifest itself as a muscle spasm(7), which makes weight bearing and hip flexion difficult and painful, eventually leading to an antalgic gait and limping. Besides radiating pain through the right thigh had caused avoidance of muscle contraction by patient which we think was the main cause for the decrease in right lower limb muscle force and a false positive FABER test.

IH is defined as calcium excretion in relation to creatinine in fasting urine samples higher than 0.80 for infants, 0.40 for preschool children, and 0.21 for children.

New researches on reductions in bone mineral density (BMD) in adults with IH in comparison with non-hypercalciuric ones, showed the possible effect of persistent hypercalciuria in decreasing bone mass and increasing risk for fracture (8-10). Lately, clinical findings indicate a similar relation between IH and bone loss in children regardless the presence of hematuria or nephrolithiasis.In our case, the diffuse osteopenia and vertebrate collapses and other bone abnormalities seem to have the same reason as in BMD decrease in IH studies. Bone loss seems mainly to involve those skeletal sites where trabecular bone is more represented, such as vertebral bodies (-). Despite the limited number of studies in children with IH, most of the results (10-12) are consistent with the view that there is a decrease in BMD, particularly at the spine, just the way it is in adults and in our case. The precise mechanisms leading and contributing to bone loss or the failure of adequate bone mass gain are yet unknown. Any continuous and persistent interference in bone mass gain may be a determining factor for low BMD, with an increased risk of osteopenia, osteoporosis and fractures in adulthood (13, 14).
